# The Role of Reticulons in Neurodegenerative Diseases

**DOI:** 10.1007/s12017-013-8271-9

**Published:** 2013-11-12

**Authors:** Valerio Chiurchiù, Mauro Maccarrone, Antonio Orlacchio

**Affiliations:** 1 Laboratorio di Neurochimica dei Lipidi, Centro Europeo di Ricerca sul Cervello (CERC) - Istituto di Ricovero e Cura a Carattere Scientifico (IRCCS) Santa Lucia, Rome, Italy; 2 Facoltà di Medicina Veterinaria, Università di Teramo, Teramo, Italy; 3 Centro Integrato di Ricerca (CIR), Università “Campus Bio-Medico” di Roma, Rome, Italy; 4 Laboratorio di Neurogenetica, Centro Europeo di Ricerca sul Cervello (CERC) - Istituto di Ricovero e Cura a Carattere Scientifico (IRCCS) Santa Lucia, Rome, Italy; 5 Dipartimento di Medicina dei Sistemi, Università di Roma “Tor Vergata”, Rome, Italy

**Keywords:** Reticulons, Neurodegenerative diseases, Endoplasmic reticulum, Membrane trafficking, Inflammation

## Abstract

Reticulons (RTNs) are a group of membrane-associated proteins mainly responsible for shaping the tubular endoplasmic reticulum network, membrane trafficking, inhibition of axonal growth, and apoptosis. These proteins share a common sequence feature, the reticulon homology domain, which consists of paired hydrophobic stretches that are believed to induce membrane curvature by acting as a *wedge* in bilayer membranes. RTNs are ubiquitously expressed in all tissues, but each RTN member exhibits a unique expression pattern that prefers certain tissues or even cell types. Recently, accumulated evidence has suggested additional and unexpected roles for RTNs, including those on DNA binding, autophagy, and several inflammatory-related functions. These manifold actions of RTNs account for their ever-growing recognition of their involvement in neurodegenerative diseases like Alzheimer’s disease, amyotrophic lateral sclerosis, multiple sclerosis, as well as hereditary spastic paraplegia. This review summarizes the latest discoveries on RTNs in human pathophysiology, and the engagement of these in neurodegeneration, along with the implications of these findings for a better understanding of the molecular events triggered by RTNs and their potential exploitation as next-generation therapeutics.

## Introduction

Reticulons (RTNs) are a super-family of proteins named after their principal subcellular localization at the endoplasmic reticulum (ER) (van de Velde et al. 1994; Oertle and Schwab 2003; Yang and Strittmatter 2007). The family originally came to prominence because of the proposed role of one member, RTN 4A, as an inhibitor of neural outgrowth and of axonal regeneration in the CNS (GrandPré et al. 2000). However, studies that are more recent have disclosed a key role of RTNs in shaping intracellular membrane-bound organelles (especially the ER) and have also highlighted the importance of these proteins in the pathogenesis of neurodegenerative diseases. Indeed, impairment of any of the processes controlled by RTNs is somehow associated with neurodegeneration. For instance, fluctuations of RTNs levels disrupt normal brain functions in several neurodegenerative disorders, as well as in schizophrenia (GrandPré et al. 2000; Yang and Strittmatter 2007). Whether our increased understanding of normal and pathological roles of RTNs makes it possible to design rational therapeutic approaches to target neurodegenerative diseases is also discussed in this review.

## From Classification to Structural Organization of RTNs

### Definition, Evolution, and Classification of RTNs

**Fig. 1:**
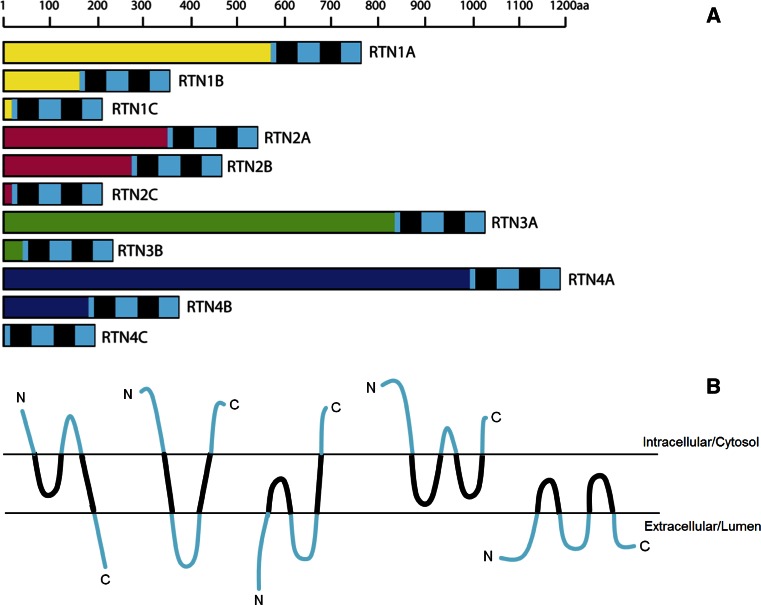
Classification and topology of mammal RTNs. **a** Schematic representation of RTNs. The *scale bar* corresponds to the length in amino acids of each RTN. The reticulon homology domain is indicated by *black* and *blue boxes*, where the *black boxes* represent the hydrophobic regions. N-Terminal regions of the different RTN isoforms are *colored*. **b** A schematic diagram illustrating the possible membrane topologies of RTNs. Based on predictions by computer programs or on biochemical assessments, RTNs may assume several topological orientations within the lipid bilayer. See text for details

Reticulons are a family of morphogenic, ER membrane-shaping proteins that stabilize highly curved ER membrane tubules. They are all characterized by the conserved reticulon homology domain (RHD), a sequence of 150–200 amino acid residues that is located at the C-terminal and is highly significant for localization and function of the protein (Oertle and Schwab 2003). All RHD-containing proteins apparently arose early in eukaryote evolution, with 4 RTN genes in mammals (RTN1–4), 2 in yeast (RTN1 and RTN2), 21 and 17 in *Arabidopsis thaliana* and *Oryza sativa* plants, respectively (Oertle et al. 2003a). Their absence in archaea or bacteria is strongly indicative of their origin in response to the evolution of endo-membrane systems. A nomenclature-based classification of RTNs has been proposed, whereby in chordates the gene and corresponding protein symbol is RTN (used throughout this manuscript), whereas in non-chordate metazoans, it is RTNL (standing for RTN-like) (Oertle et al. 2003a). In many species, multiple RTN genes are present, and these appear to have stemmed from duplications from a single common ancestor (Bandtlow et al. 2004). The four mammalian genes (RTN1–4) generate at least 11 splice variants. RTN1 yields two major proteins, called RTN1A and RTN1C, and, to a lesser extent, a third RTN1B variant. RTN2 gives rise to 3 proteins, called RTN2A, RTN2B, and RTN2C, whereas RTN3 generates RTN3A and RTN3B. Finally, RTN4 yields 3 major isoforms (RTN4A, RTN4B, and RTN4C), also referred to as NogoA, NogoB, and NogoC (Roebroek et al. 1993; van de Velde et al. 1994; Geisler et al. 1998; Roebroek et al. 1998; Moreira et al. 1999; Yang et al. 2000) (Fig. 1a).

### The RHD and RTN Membrane Topology

The key feature of the RHD is the presence of two unusually long hydrophobic regions (Fig. 1b), separated by a 66 amino acid long, hydrophilic loop (also known as Nogo66 loop), and followed by a short C-terminal tail (van de Velde et al. 1994; Oertle and Schwab 2003; Yang and Strittmatter 2007). The presence of a conserved RHD is responsible for the commonality among functions of distinct RTNs and for their subcellular localization and interaction with other proteins. Intriguingly, the fact that RTNs do not display the canonical N-terminal ER-localization signal, and the relatively longer sequence of the two hydrophobic regions (~30–35 amino acids, compared to a typical ~20 amino acids transmembrane domain of), suggests that RTN retention within ER membranes could be mainly due to the structural conformation or topology of RHD (van de Velde et al. 1994; Oertle and Schwab 2003; Yang and Strittmatter 2007). This hypothesis led to early speculations that the hydrophobic domains might “double back” within the membrane, forming hairpin loops into the outer leaflet of the lipid bilayer responsible for membrane bending (Oertle and Schwab 2003). However, other conformations are possible and, based on predictions by in silico analysis, RTNs may adopt a different folding which is able to flip–flop within the lipid bilayer due to the presence of charged residues within the two hydrophobic domains (Fig. 1b). These domains are indeed long enough to loop back within the lipid bilayer, thus giving rise to several alternative orientations of RTNs (Oertle et al. 2003a, b, c; Voeltz et al. 2006; He et al. 2007; Sparkes et al. 2010). Although there is some evidence that RHD membrane topology can be influenced by protein sequence in the hydrophilic loop and in the N-terminal domain (He et al. 2007), future biochemical studies are deemed necessary to determine the actual topology of RTNs and the identity of factors that can affect it.

### N-Terminal Domain of Mammalian RTNs

In contrast to RHD, N-terminal regions of RTNs are not conserved and show dramatic differences in terms of length and sequence, even within RTNs isoforms (Oertle and Schwab 2003; Yang and Strittmatter 2007). In some RTNs, like RTN1C, RTN2C, and RTN4C, the N-terminal sequences are extremely short, with the bulk of the protein mainly consisting of RHD. In others, like RTN1A/B, RTN2A/B, RTN3A, and RTN4A/B, the N-terminal regions are much larger and are likely to confer specific biological functions. Somewhat surprisingly, no recognized protein domains have been identified so far in N-terminal regions, whose function remains mostly unknown. However, N terminus of RTN4A and RTN4B is rich in proline and contains large unstructured regions (Li and Song 2007). Furthermore, N terminus of all RTN4 isoforms lacks a specific signal sequence for ER translocation, accounting for their presence both in the ER and at the cell surface. The presence of such unstructured N-terminal domains in RTN4A/B allows them to form multiprotein structures and to carry out alternative functions. A role in mediating the interaction with other functional partners has been documented and may contribute to the facilitation of the binding of Nogo66 to its receptor (as explained later) (Hu et al. 2005) or the degradation of RTNs. Indeed, phosphorylation of the N terminus of RTN4B by cyclin-dependent kinases 1 and 2 renders the protein susceptible to cleavage by caspase-7 (Schweigreiter et al. 2007).

### RTNs as Mediators of Physiological Functions

**Fig. 2:**
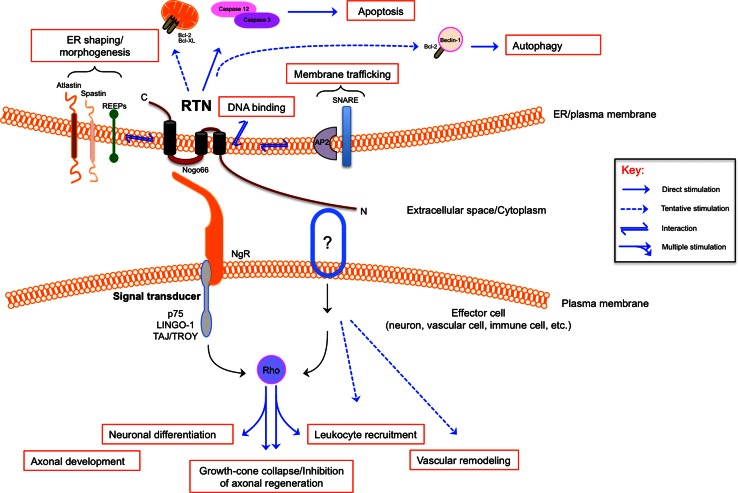
Diverse functions and molecular mechanisms of RTNs. The hydrophobic domains of RTNs can interact with other transmembrane proteins involved in ER shaping and morphogenesis (REEPs, atlastins, spastin) and membrane trafficking (SNARE, AP2), they can directly bind DNA or regulate apoptosis and autophagy through caspases activation and control of Bcl-2-family proteins. The majority of the diverse functions of RTNs occur through the interaction of the hydrophilic loop Nogo66 with its receptor NgR, or by the interaction of their N-terminal tail with a purported receptor. In case of RTN4, these interactions mostly lead to Rho signaling activation. See text for details

The ubiquitous expression of RTNs suggests a general role in the functioning of this organelle. However, although ubiquitously expressed in all tissues, each individual RTN can still exhibit a degree of tissue- or cell-specific pattern (Oertle and Schwab 2003). Furthermore, many cell types express more than one RTN, suggesting that some of these proteins may have additional specific functions beyond those at the ER. In the following sections, we shall outline what is known about common and distinct roles of RTNs (Fig. 2), except for RTN2 for which no specific activity has been unveiled yet. Commonalities and specificities of RTNs in human pathophysiology are also summarized in Table 1.

**Table 1:** Role of RTNs in human pathophysiology

Reticulon	Localization	Common functions	Specific functions	Neurodegenerative diseases
RTN1 (Wieczoerk and Hughes 1991; Roebroek et al. 1993; van de Velde et al. 1994)	Brain Neuroendocrine tissues	ER shaping/morphogenesis Membrane trafficking Apoptosis	Neuronal differentiation DNA binding/epigenetic modifications	?
RTN2 (Geisler et al. 1998; Roebroek et al. 1998)	Brain Muscle		?	Hereditary spastic paraplegia-12
RTN3 (Moreira et al. 1999; Cai et al. 2005)	Brain Olfactory nerve		Axonal development Autophagy	Alzheimer’s disease
RTN4 (Chen et al. 2000; GrandPré et al. 2000; Bi et al. 2000)	Brain Leukocytes Vasculature Skin		Inhibition of neurite and axonal growth Leukocyte recruitment Vascular remodeling	Alzheimer’s disease Amyotrophic lateral sclerosis Multiple sclerosis

### Common Functions of RTNs

#### ER Shaping and Morphogenesis

A major advance in our understanding of the function of RTNs came with the identification of members of the RTN family as key proteins involved in shaping and morphogenesis of the ER. This role of the RTNs has been recently discussed in extensive reviews (Shibata et al. 2009; Hu et al. 2011; Park and Blackstone 2010), thus here we shall briefly summarize it. RTNs interact with members of another class of proteins, the DP1/yop1p/REEP family (referred to as REEPs throughout this article) (Voeltz et al. 2006). Both RTNs and REEPs localize at the highly curved regions of ER tubules, and in many species, their cellular depletion causes defects in the formation of the tubular ER; conversely, their overexpression leads to increased ER tubulation (Voeltz et al. 2006; Sparkes et al. 2010; O’Sullivan et al. 2012). In addition, in vitro reconstitution experiments have shown that RTN (e.g., Rtn1p) or REEP (e.g., Yop1p) is sufficient to tubulate proteoliposomes (Hu et al. 2008). Similarly, to RTNs, REEPs have a conserved double hairpin membrane domain that is critical to confer the ability to shape ER tubules (Voeltz et al. 2006; Hu et al. 2008; Zurek et al. 2011). These observations have led to a model for ER tubule formation whereby RTNs and REEPs act synergistically to trigger and stabilize membrane curvature by hydrophobic wedging. In this process, the hairpin membrane domains displace lipids from the outer layer better than from the inner layer (Hu et al. 2011). It has been proposed that oligomerized RTNs and REEPs act as a scaffold to impose a cylindrical structure on the membrane curvature, thus generating a tubule (Hu et al. 2008). By a similar mechanism, the same proteins are also involved in generating the highly curved edges of ER sheets (Shibata et al. 2010). Two additional classes of proteins act in concert with RTNs and REEPs during ER morphogenesis: atlastins, large GTPases that mediate homotypic fusion of ER tubules, and spastin, an ATPase that separates microtubules, particularly in relation to membrane modeling events (Errico et al. 2002; Orlacchio et al. 2004; Connell et al. 2009; Hu et al. 2009; Bian et al. 2011; Morin-Leisk et al. 2011; Moss et al. 2011; Liu et al. 2012; Lumb et al. 2012). In line with this, the expression of an ATPase-defective spastin results in extensive tubulation of the ER, highlighting its role as a microtubule-severing protein during ER shaping (Connell et al. 2009). Both atlastins and spastin possess long hydrophobic domains that are proposed to act as *hairpin wedges*, and both interact with RTNs and REEPs as well as with each other (Sanderson et al. 2006; Park et al. 2010; Montenegro et al. 2012).

#### Membrane Trafficking

Although RTNs are principally located within ER membranes, they have also been described at the level of Golgi and plasma membranes, suggesting that they may act also at these sites. In this context, it is interesting that several studies suggest that RTNs may be involved in the secretory pathway. For example, RTN1C co-immunoprecipitates with a variety of SNARE proteins that are engaged in regulated secretion, and ectopic expression of a fragment of RTN1C in PC12 cells leads to an increased rate of growth hormone release (Steiner et al. 2004). Similarly, RTN2B is a positive regulator of the delivery of the neuronal EAAC1 glutamate transporter from the ER to the cell surface, via a mechanism that appears to facilitate exit of the transporter from ER (Liu et al. 2008). RTN3 has been implicated in early phases of the secretory pathway, because its overexpression in HeLa cells interferes with both anterograde and retrograde transport between ER and Golgi (Wakana et al. 2005). A caveat is that often the functional data rely on overexpression studies, but they lack evidence on the role of constitutive proteins under more physiological conditions. Therefore, additional experiments should be performed to better assess the in vivo relevance of these findings.

#### Apoptosis

Several lines of evidence indicate that RTNs are involved in cell death pathways, most notably in ER stress-induced apoptosis. Interestingly, RTN1C or RTN4A/B overexpression induces *per se* apoptosis by inhibiting the apoptotic inhibitors Bcl-XL and/or Bcl-2; instead, RTN1C, RTN3, or RTN4B overexpression causes ER stress-mediated apoptosis paralleled by Ca^2+^ depletion from ER stores (Tagami et al. 2000; Kuang et al. 2005; Kuang et al. 2006; Tufi et al. 2008). Consistently, RTN1C-overexpressing neuroblastoma cells show a significant increase in their susceptibility to cell death upon ER stress induction (Di Sano et al. 2007). In addition, in HeLa cells, RTN3 causes the activation of caspases-3 and caspases-12, along with the collapse of mitochondrial membrane potential and the release of cytochrome c (Xu et al. 2006). RTN3 also enhances TRAIL-induced apoptosis through the induction of DR5 expression in human renal cancer cells (Lee et al. 2009). However, literature data on the role of RTNs in apoptosis are partly controversial, inasmuch as not only RTN4B may exert anti-apoptotic functions (Oertle et al. 2003b; Schweigreiter et al. 2007), but ER stress-mediated RTN3 up-regulation can also be protective against several insults by contributing to Bcl-2 translocation to the mitochondria (Wan et al. 2007). Indeed, RTN3 can interact with Bcl-2 both in vitro and in vivo and can mediate its accumulation in mitochondria, thus modulating its anti-apoptotic activity (Zhu et al. 2007).

### Distinct Roles of RTNs

#### RTN1

The first member of the RTNs family to be identified in 1993 is mainly expressed in neurons and cells of neuroendocrine tissues (Roebroek et al. 1998). The RTN1C isoform is the most studied and has long been considered a marker of neuronal differentiation because its expression is increased upon differentiation in several neuroblastoma cell lines (Wieczoerk and Hughes 1991; Hens et al. 1998). In the C-terminal region of RTN1C, the GAKRH sequence (a signature of H4 histone) confers a strong ability to bind and condense DNA. Such a binding activity of RTN1C is regulated by acetylation/deacetylation and is coupled to inhibition of HDAC activity (Fazi et al. 2009; Melino et al. 2009; Nepravishta et al. 2010). The apparently unique ability of RTN1 to affect epigenetic modifications is believed to play a major role in its biological functions. The first clues for a functional role of N-terminal domains of RTN1 isoforms came very recently from two interesting studies. On one hand, it was reported that RTN1C was able to modulate the nitrosylation-dependent activity of protein disulfide isomerase, which is involved in cellular defense against ER stress-related protein misfolding (Bernardoni et al. 2013). Kaya et al. (2013) identified the intracellular calcium release channel ryanodine receptor 2 (RyR2) as a novel binding partner of RTN1A in neurons both in vitro and in vivo and showed a RTN1A-dependent inhibitory effect on the RyR2 activity, with potential implication in neurotransmitter release and in aging-related Ca^2+^ dysregulation.

#### RTN3

It was firstly isolated from the retina and later shown to have a widespread expression, with the highest levels in the brain (Moreira et al. 1999; Qi et al. 2003; Cai et al. 2005). RTN3 expression in the optic and olfactory nerve during both developmental and adult stages, along with its co-localization with synaptophysin in the tubulovesicular structures in the developing axon of cultured cortical neurons, suggested an important role in axonal development (Kumamaru et al. 2004). Moreover, RTN3 has been recently linked to autophagy, because its depletion increased autophagy induction in human prion-overexpressing neuroblastoma cells, with subsequent enhancement of the clearance of prion aggregates (Chen et al. 2011). In particular, RTN3 inhibited Beclin-1-dependent autophagy by promoting the formation of the inactive Bcl-2/Beclin-1 complex (Chen et al. 2011). Yet, there is no evidence that other RTNs, despite they also interact with Bcl-2, may be implicated in autophagy. In yeast, combined deletion of a RTN with the REEP homolog yop1p leads to inhibition of the replication of positive-strand RNA viruses (Diaz et al. 2010). The latter replicate in curved intracellular membrane compartments that are absent when membrane-shaping proteins are defective. In support of the idea that membrane shaping by RTNs is important in viral replication, studies in mammalian cells have shown that RTN3 interacts with viral replication components and is necessary and sufficient for enteroviral replication (Tang et al. 2007). Recently, the role of RTN3 has also been associated with neuritic dystrophy and cognitive deficits, with particular reference to diabetic-induced dementia (Shi et al. 2013; Deng et al. 2013; Zhao et al. 2013).

#### RTN4

Commonly known as Nogo, it is undoubtedly the most studied member of the RTN family. The RTN4 isoforms RTN4A, RTN4B, and RTN4C have very different distribution patterns throughout the nervous system, as well as in peripheral body districts. RTN4A is largely, but not exclusively, expressed in the nervous system, in particular in cerebral cortex, hippocampus, diencephalon, cerebellum, and spinal cord. Outside the CNS, RTN4A is expressed in skin, skeletal muscle, heart as well as in certain immune cells, in particular macrophages (Schwab 2010). RTN4B is expressed in many tissues, including the central and peripheral nervous systems, as well as at several peripheral sites such as vascular endothelial cells and inflammatory cells (Acevedo et al. 2004; Schwab 2010). RTN4C expression, instead, is poorly documented, mainly due to the lack of human-specific antibodies and probes. However, it is highly expressed in skeletal muscle, in some types of neurons of rat CNS, such as cerebellar Purkinje cells, and weakly expressed in liver and kidney (Josephson et al. 2001; Schwab 2010). Ample evidence has supported RTN4 (especially the RTN4A isoform) as an important agent in inhibiting neurite outgrowth and axonal regeneration (Chen et al. 2000; GrandPré et al. 2000; Prinjha et al. 2000; Kim et al. 2003; Oertle et al. 2003c; Reindl et al. 2003; Simonen et al. 2003; Karnezis et al. 2004). The role of RTN4 in these processes has been extensively reviewed and will be briefly summarized here. The current model suggests that the extracellular hydrophilic Nogo66 loop is expressed on the surface of oligodendrocytes or Schwann cells and binds to an axonal Nogo66 receptor (NgR) (O’Neill et al. 2004). Interestingly, since NgR is localized on the outer layer of the plasma membrane and lacks a transmembrane domain, its signaling cascade engages several transducers and is strictly dependent on downstream activation of Ras homologous gene family, member A (Rho-A) (Ellezam et al. 2002; Fournier et al. 2003). These signaling transducers include the neurotrophin receptor p75, the transmembrane protein LINGO-1, the orphan tumor necrosis factor family member TAJ/TROY (Wang et al. 2002; Mi et al. 2005; Shao et al. 2005). Of notice, several studies have recently reported that Nogo proteins play a crucial role in inflammation-related processes, most of them linked to leukocyte recruitment at sites of acute or chronic inflammation (Acevedo et al. 2004; Yu et al. 2009; Wright et al. 2010; Di Lorenzo et al. 2011; Schanda et al. 2011), inhibition of endothelial cell migration (Wälchli et al. 2013), liver pathophysiology (Zhang et al. 2011; Gao et al. 2013), as well as regulation of synaptic plasticity and cognitive functions (Tews et al. 2013; VanGuilder Starkey et al. 2013). These novel findings seem to be independent of the RHD motif; yet, the underlying molecular events are likely to rely on the NgR-induced RhoA pathway and on the reported capability of Nogo proteins to associate with cytoskeletal structures in immune cells (Schanda et al. 2011). Interestingly, leukocytes extravasation across the blood–brain barrier and their subsequent accumulation at sites of injury is now recognized as a hallmark of neurodegenerative diseases (Rezai-Zadeh et al. 2009; Schwartz et al. 2009).

## The Role of RTNs in the Pathological Processes of Neurodegenerative Diseases

### Alzheimer’s Disease

Alzheimer’s disease (AD) is probably the most common neurodegenerative disease and, although its etiology is still unclear, it is characterized by the presence of brain amyloid plaques and neurofibrillary tangles whose accumulation ultimately leads to extensive neuronal loss and progressive decline of cognitive function (Chiurchiù and Maccarrone 2011; Huang and Mucke 2012). All 4 human RTN proteins seem to be involved in AD, inasmuch as beta-site amyloid precursor protein-cleaving enzyme1 (BACE1), the β-secretase responsible for the cleavage of the amyloid precursor protein (APP) into β-amyloid peptide (Aβ) was found to co-immunoprecipitate with RTN1, RTN2, RTN3, and RTN4 (He et al. 2004). Yet, research efforts have been focused mainly on RTN3 and RTN4, which were shown to interact with BACE1, most likely through the RHD (He et al. 2004; Murayama et al. 2006). *In* *vitro* overexpression of RTN3 reduced the levels of Aβ produced by HEK-293 cells, and conversely, knockdown of RTN3 by RNA interference increased Aβ levels, consistent with the idea that RTN3 inhibits BACE1. Notably, in a subtractive hybridization screening, human RTN3 was found to be downregulated in the temporal lobes of AD patients (Yokota et al. 2006). Although the two-transmembrane-domain tertiary structure of RTN3 and RTN4 seems to critically affect BACE1 enzymatic activity (Kume et al. 2009a, b), the relevance of these RTNs for AD pathology and their exact role remains controversial and thus yet to be established. On the one hand, a mechanism by which RTN3 may function as a negative modulator of Aβ production has been proposed and is such that the association of RTN3–BACE1 may alter its trafficking itinerary, thus affecting BACE1 enzymatic activity more markedly in post-Golgi compartments like the endosomes (Small and Gandy 2006; Shi et al. 2009). On the other hand, RTN3 seems to act as a marker for AD, inasmuch as it has been shown to oligomerize and accumulate in a subpopulation of dystrophic neuritis in AD postmortem brain (Hu et al. 2007). Consistently, transgenic mice overexpressing RTN3 develop dystrophic neuritis that are morphologically similar to those observed in AD brains and that correlate with the formation of RTN3 aggregates in susceptible brain regions (Hu et al. 2007). However, these findings are in conflict with those by Kume et al. (2009a, b), in which, although reporting that RTN3 subcellularly co-localizes with BACE1, no significant differences in its expression levels were observed between control and AD brains.

### Amyotrophic Lateral Sclerosis

Amyotrophic lateral sclerosis (ALS) is a fatal neurodegenerative disease characterized by the death of both upper and lower motor neurons of the brain, brain stem, and spinal cord, overall leading to progressive weakness and atrophy of skeletal muscles (Mitchell and Borasio 2007; Chiurchiù and Maccarrone 2011). Both in postmortem muscular samples of ALS patients and in ALS mouse models expressing human superoxide dismutase (SOD) with a disease-causing dominant mutation, differential up- and down-regulation of RTN4A/B and RTN4C mRNA compared with wild-type mice was reported (Dupuis et al. 2000). Levels of RTN4A (and to a lesser extent of RTN4B) mRNA increased in pre-symptomatic animals, whereas RTN4C was highly expressed in asymptomatic mice only (Dupuis et al. 2000). Indeed, the levels of RTN4A and RTN4B, which are barely detectable in adult muscles, increased in ALS mice and correlated with disease severity (Jokic et al. 2005), whereas the levels of the highly expressed RTN4C isoform decreased (Dupuis et al. 2002). Beyond these expression and mechanistic studies, several functional evidences also emerged. For instance, a link between the early overexpression of RTN4A in ALS muscle fibers was found and consisted in an impairment of the neuromuscular junction, ultimately leading to motor neuron degeneration (Jokic et al. 2006). Additionally, RTN4A was protected from SOD1-dependent ASL by contributing to the correct functioning of the ER (Yang et al. 2009). Conversely, several reports indicate that RTN4A expression is not unique to this disorder (Wojcik et al. 2006; Pradat et al. 2007; Harel et al. 2009; Askanas et al. 2007). Overall, there is a general consensus that we have to be cautions as to consider RTN4 as a specific biomarker for ALS, thus questioning whether this protein could be a possible candidate target for novel drugs against the disease (Tågerud et al. 2007).

### Multiple Sclerosis

Multiple sclerosis (MS) is a chronic inflammatory, progressive, and degenerative disorder characterized by intermittent episodes of demyelination and axonal loss or damage in the CNS. Although its etiology has not been fully elucidated yet, it is likely that both genetic and environmental components play a crucial role in disease onset and progression, and it is now well recognized that immunological mechanisms are the initial trigger of MS (Noseworthy et al. 2000; Chiurchiù and Maccarrone 2011; Chiurchiù et al. 2013). Autoantibodies against RTN4A have been found in serum and cerebrospinal fluid of patients with MS, especially in those with relapsing-remitting rather than chronic progressive MS (Reindl et al. 2003). Interestingly, administration of exogenous anti-RTN4A antibodies protected against demyelination in the experimental autoimmune encephalomyelitis (EAE) mouse model of MS, inasmuch as it suppressed inflammatory responses in these animals (Karnezis et al. 2004). Consistently, RTN4 knockout mice showed a significantly delayed onset of EAE development, and passive immunization with anti-Nogo immunoglobulin suppressed inflammatory processes associated with EAE (Karnezis et al. 2004). Given the importance of RTN4 in neurite growth within the brain, these data suggest that its blockade could be beneficial to preserve or perhaps even restore neuronal integrity after demyelination and axonal loss or damage during MS. However, vaccination with Nogo66-derived peptides may prevent but also promote encephalitogenic reactions, depending on the type of the recruited immune response (Fontoura et al. 2004). Furthermore, analyses of demyelinating lesions in MS patients demonstrated that RTN4A is highly expressed in oligodendrocytes, while its NgR receptor appears specifically in reactive astrocytes and microglia/macrophages (Satoh et al. 2005). The biological meaning of this distinct expression pattern remains unknown, but it could influence disease pathogenesis and future therapeutic approaches. Indeed recent evidence supports the concept that targeting RTN4 signaling in MS could be a promising therapeutic approach (Lee and Petratos 2013). In fact, impairment of LINGO-1 or blockade of its pathway, as well as silencing of RTN4A, promoted functional recovery and limited clinical severity by stimulating axonal growth and repair in MOG_35–55_- and myelin basic protein-induced EAE models, ultimately improving the functional deficits caused by immune-mediated axonal damage (Mi et al. 2007; Yang et al. 2010). The potential mechanism of such therapeutic effect likely involves a reduction in the NgR-dependent signaling which limited the activation of the phosphorylation of CRMP-2, a crucial tubulin-associated protein that regulates axonal growth, thus preventing axonal degeneration and neurological decline (Petratos et al. 2012).

### Hereditary Spastic Paraplegia

**Fig. 3:**
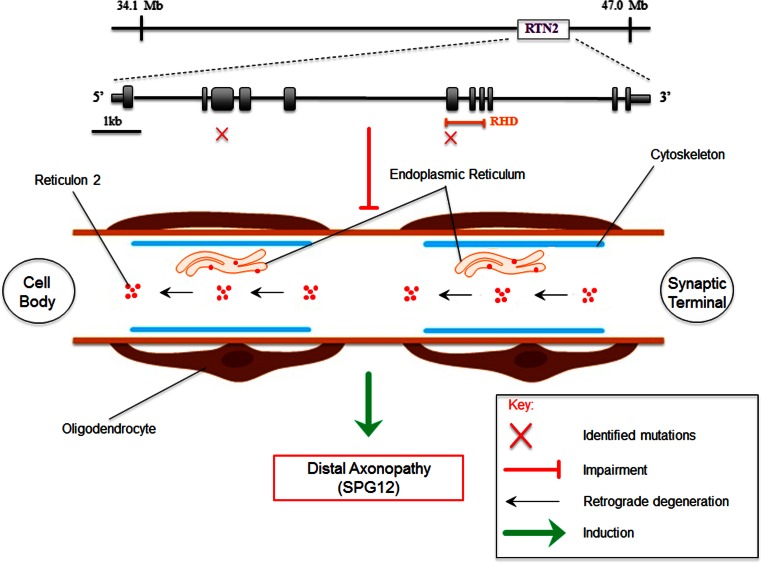
Role of RTN2 in HSP. A schematic of the *RTN2* gene is drawn to scale with the corresponding conserved protein domains and the identified mutations (*red crosses*). Haploinsufficiency of RTN2 cause the autosomal dominant spastic paraplegia 12 (SPG12) and is a likely mechanism for the injury of the distal axon (axonopathy) with subsequent retrograde or “dying back” axonal degeneration toward the neuronal cell body. See text for details

Hereditary spastic paraplegia (HSP) includes a large and diverse group of genetic disorders whose main feature is progressive spasticity and weakness in the lower limbs, as a result of continuous distal axonopathy caused by defects in the mechanisms that transport proteins and substances along the axons (Fink 2013; Blackstone et al. 2011; Blackstone 2012). At least four autosomal dominant HSP are caused by mutations in genes encoding proteins involved in ER morphogenesis and that bear an intramembrane hairpin loop responsible for the curvature of ER membranes and for their reciprocal interactions. These include spastin, the most commonly mutated protein in HSP, atlastin-1, REEP1, and RTN2 (Wakana et al. 2005; Orso et al. 2009; Blackstone 2012). Not surprisingly, several members of the RTN family have been shown to interact with the latter proteins. Indeed, RTN3 and RTN4 interact with atlastin-1 (Hu et al. 2009), whereas RTN1 and RTN3 have been found to interact with spastin (Mannan et al. 2006a, b). The evidence that mutations in the RTN2 gene cause the autosomal dominant spastic paraplegia 12 (SPG12) was very recently demonstrated by our group (Montenegro et al. 2012). We have also shown that RTN2 interacts with spastin, an interaction that requires the predicted hairpin membrane domain in the latter protein. Thus, unlike other RTNs, whose involvement in neurodegenerative diseases is still at a mechanistic level, this is the first genetic evidence that a member of the RTNs family directly causes a specific disorder, with haploinsufficiency of RTN2 likely being the molecular pathological mechanism for axonopathy (Fig. 3). In keeping with this, the *Drosophila melanogaster* ortholog of the HSP gene *RTN2* (called *RTN*-*like 1* gene) was found to be required for organization of ER and of distal motor axons, thus supporting the concept that HSP can be caused by axonal ER impairment (O’Sullivan et al. 2012). These results demonstrate that RTN2 protein participates in the network of HSP proteins involved in ER shaping and provide further direct evidence not only that correct ER shaping may be important in general axonal maintenance, but also that abnormal ER morphogenesis is a potential pathogenic mechanism in HSP. These findings will set the basis for future studies aimed at determining how abnormal ER morphogenesis causes axonal degeneration.

## Concluding Remarks and Future Directions

The engagement of RTNs in neurodegeneration is a rather challenging issue, because of the large number of isoforms of each RTN, and the fact that all of these proteins are almost equally expressed within the brain, where they share common functions. This scenario is further complicated by the yet unclear molecular events triggered by RTNs. Nonetheless, although the mechanism by which abnormal ER morphogenesis can cause degeneration of neuronal cells is unknown, the crucial role of these proteins in ER functioning is apparent and it is not surprising that most of neurodegenerative disorders are related to ER-associated dysfunctions, including protein misfolding and cell death. Of note, the concept that in biology the same protein acquires multiple roles during evolution holds true also in the case of RTNs, which initially emerged as a family of neuroendocrine-specific proteins whose function was only attributed to their RHD topology and are now being recognized as modulators of several other functions possibly alleged to the N-terminal domain. The recent findings of a role for RTNs in the modulation of vascular and immune functions appear to be consistent with this view, as the isoforms which bear the longest N-terminal regions are indeed the most active in these processes. Future studies focused on the role of each RTN protein in distinct peripheral regions of the body, in health and disease, will provide valuable insights into their expanding role in both neurodegeneration and inflammation. Furthermore, the emerging findings summarized here suggest the existence of a complex RTN signaling network that affects immune functions by engaging NgR or a yet unidentified N-terminal receptor. Thus, additional studies on the signaling impact of N-terminal regions of RTNs are needed in order to provide deeper insights into the roles of their ancient receptors in the generation, plasticity, and pathology of the nervous system, as well as in immune-mediated processes typical of neurodegenerative diseases. Accordingly, the different RTN expression patterns between healthy and diseased tissues could represent one of the most interesting keys for designing new therapeutics. However, an RTN-based pharmacological approach is far from being on the way, because many questions concerning the exact role of RTNs await to be answered, and further investigations are needed to confirm whether these proteins may be potential therapeutic targets in neurodegenerative diseases. For instance, one difficulty in designing appropriate drugs that target RTN3 in AD is the fact that both increases and decreases of this protein have been reported in this disorder. Furthermore, future pharmacological strategies should also take into account the manifold physiological functions of RTNs in order to provide an effective therapeutic treatment. The genetic correlation of RTN2 in the induction of SPG12 makes it the only member of the RTNs family that could hitherto be potentially targeted. On a final note, the discovery that RTN1C is a DNA-binding protein seems of particular interest, because it adds the new dimension of epigenetic regulation of gene expression to the already complete machinery involved in neurodegenerative processes.

## References

[CR1] Acevedo L, Yu J, Erdjument-Bromage H, Kim JE, Fulton D, Tempst P (2004). A new role for Nogo as a regulator of vascular remodeling. Nature Medicine.

[CR2] Askanas V, Wojcik S, Engel WK (2007). Expression of Nogo-A in human muscle fibres is not specific for amyotrophic lateral sclerosis. Annals of Neurology.

[CR3] Bandtlow CE, Dlaska M, Pirker S, Czech T, Baumgartner C, Sperk G (2004). Increased expression of Nogo-A in hippocampal neurons of patients with temporal lobe epilepsy. European Journal of Neuroscience.

[CR4] Bernardoni P, Fazi B, Costanzi A, Nardacci R, Montagna C, Filomeni G (2013). Reticulon1-C modulates protein disulphide isomerase function. Cell Death & Disease.

[CR5] Bi A, Yu L, Yang J, Zhang M, Zhou Y, Zhao S (2000). Cloning and expression analysis of human reticulon 4c cDNA. Chinese Science Bulletin.

[CR6] Bian X, Klemm RW, Liu TY, Zhang M, Sun S, Sui X (2011). Structures of the atlastin GTPase provide insight into homotypic fusion of endoplasmic reticulum membranes. Proceedings of the National Academy Sciences of the United States of America.

[CR7] Blackstone C (2012). Cellular pathways of hereditary spastic paraplegia. Annual Review of Neuroscience.

[CR8] Blackstone C, O’Kane CJ, Reid E (2011). Hereditary spastic paraplegias: Membrane traffic and the motor pathway. Nature Review Neuroscience.

[CR9] Cai Y, Saiyin H, Lin Q, Zhang P, Tang L, Pan X (2005). Identification of a new RTN3 transcript, RTN3-A1, and its distribution in adult mouse brain. Molecular Brain Research.

[CR10] Chen MS, Huber AB, van der Haar ME, Frank M, Schnell L, Spillmann AA (2000). Nogo-A is a myelin-associated neurite outgrowth inhibitor and an antigen for monoclonal antibody IN-1. Nature.

[CR11] Chen R, Jin R, Wu L, Ye X, Yang Y, Luo K (2011). Reticulon 3 attenuates the clearance of cytosolic prion aggregates via inhibiting autophagy. Autophagy.

[CR1200] Chiurchiù, V., Cencioni, M. T., Bisicchia, E., De Bardi, M., Gasperini, C., Borsellino, G., et al. (2013). Distinct modulation of human myeloid and plasmacytoid dendritic cells by anandamide in multiple sclerosis. *Annals of Neurology, 73*(5), 626–636. 10.1002/ana.23875 23447381

[CR12] Chiurchiù V, Maccarrone M (2011). Chronic inflammatory disorders and their redox control: From molecular mechanisms to therapeutic opportunities. Antioxidants & Redox Signaling.

[CR13] Connell JW, Lindon C, Luzio JP, Reid E (2009). Spastin couples microtubule severing to membrane traffic in completion of cytokinesis and secretion. Traffic.

[CR14] Deng M, He W, Tan Y, Han H, Hu X, Xia K (2013). Increased expression of reticulon 3 in neurons leads to reduced axonal transport of β-site amyloid precursor protein cleaving enzyme 1. Journal of Biological Chemistry.

[CR15] Di Lorenzo A, Manes TD, Davalos A, Wright PL, Sessa WC (2011). Endothelial reticulon-4B (Nogo-B) regulates ICAM-1-mediated leukocyte transmigration and acute inflammation. Blood.

[CR16] Di Sano F, Fazi B, Tufi R, Nardacci R, Piacentini M (2007). Reticulon-1C acts as a molecular switch between endoplasmic reticulum stress and genotoxic cell death pathway in human neuroblastoma cells. Journal of Neurochemistry.

[CR17] Diaz A, Wang X, Ahlquist P (2010). Membrane-shaping host reticulon proteins play crucial roles in viral RNA replication compartment formation and function. Proceedings of the National Academy Sciences of the United States of America.

[CR18] Dupuis L, de Tapia M, René F, Lutz-Bucher B, Gordon JW, Mercken L (2000). Differential screening of mutated SOD1 transgenic mice reveals early up-regulation of a fast axonal transport component in spinal cord motor neuron. Neurobiology of Disease.

[CR19] Dupuis L, Gonzalez de Aguilar JL, di Scala F, Rene F, de Tapia M, Pradat PF (2002). Nogo provides a molecular marker for diagnosis of amyotrophic lateral sclerosis. Neurobiology of Disease.

[CR20] Ellezam B, Dubreuil C, Winton M, Loy L, Dergham P, Sellés-Navarro I (2002). Inactivation of intracellular Rho to stimulate axon growth and regeneration. Progress in Brain Research.

[CR21] Errico A, Ballabio A, Rugarli EI (2002). Spastin, the protein mutated in autosomal dominant hereditary spastic paraplegia, is involved in microtubule dynamics. Human Molecular Genetics.

[CR22] Fazi B, Melino S, De Rubeis S, Bagni C, Paci M, Piacentini M (2009). Acetylation of RTN-1C regulates the induction of ER stress by the inhibition of HDAC activity in neuroectodermal tumors. Oncogene.

[CR23] Fink JK (2013). Hereditary spastic paraplegia: Clinico-pathologic features and emerging molecular mechanisms. Acta Neuropathologica.

[CR24] Fontoura P, Ho PP, DeVoss J, Zheng B, Lee BJ, Kidd BA (2004). Immunity to the extracellular domain of Nogo-A modulates experimental autoimmune encephalomyelitis. The Journal of Immunology.

[CR25] Fournier AE, Takizawa BT, Strittmatter SM (2003). Rho kinase inhibition enhances axonal regeneration in the injured CNS. The Journal of Neuroscience.

[CR26] Gao L, Utsumi T, Tashiro K, Liu B, Zhang D, Swenson ES, Iwakiri Y (2013). Reticulon 4B (Nogo-B) facilitates hepatocyte proliferation and liver regeneration in mice. Hepatology.

[CR27] Geisler JG, Stubbs LJ, Wasserman WW, Mucenski ML (1998). Molecular cloning of a novel mouse gene with predominant muscle and neural expression. Mammalian Genome.

[CR28] GrandPré T, Nakamura F, Vartanian T, Strittmatter SM (2000). Identification of the Nogo inhibitor of axon regeneration as a reticulon protein. Nature.

[CR29] Harel NY, Cudkowicz ME, Brown RH, Strittmatter SM (2009). Serum Nogo-A levels are not elevated in amyotrophic lateral sclerosis patients. Biomarkers.

[CR30] He W, Lu Y, Qahwash I, Hu XY, Chang A, Yan R (2004). Reticulon family members modulate BACE1 activity and amyloid-beta peptide generation. Nature Medicine.

[CR31] He W, Shi Q, Hu X, Yan R (2007). The membrane topology of RTN3 and its effect on binding of RTN3 to BACE1. The Journal of Biological Chemistry.

[CR32] Hens J, Nuydens R, Geerts H, Senden NH, Van de Ven WJ, Roebroek AJ (1998). Neuronal differentiation is accompanied by NSP-C expression. Cell and Tissue Research.

[CR33] Hu F, Liu BP, Budel S, Liao J, Chin J, Fournier A (2005). Nogo-A interacts with the Nogo-66 receptor through multiple sites to create an isoform-selective subnanomolar agonist. The Journal of Neuroscience.

[CR34] Hu J, Prinz WA, Rapoport TA (2011). Weaving the web of ER tubules. Cell.

[CR35] Hu X, Shi Q, Zhou X, He W, Yi H, Yin X (2007). Transgenic mice overexpressing reticulon 3 develop neuritic abnormalities. EMBO Journal.

[CR36] Hu J, Shibata Y, Voss C, Shemesh T, Li Z, Coughlin M (2008). Membrane proteins of the endoplasmic reticulum induce high-curvature tubules. Science.

[CR37] Hu J, Shibata Y, Zhu PP, Voss C, Rismanchi N, Prinz WA (2009). A class of dynamin-like GTPases involved in the generation of the tubular ER network. Cell.

[CR38] Huang Y, Mucke L (2012). Alzheimer mechanisms and therapeutic strategies. Cell.

[CR39] Jokic N, Gonzalez de Aguilar JL, Dimou L, Lin S, Fergani A, Ruegg MA (2006). The neurite outgrowth inhibitor Nogo-A promotes denervation in an amyotrophic lateral sclerosis model. EMBO Reports.

[CR40] Jokic N, Gonzalez de Aguilar JL, Pradat PF, Dupuis L, Echaniz-Laguna A, Muller A (2005). Nogo expression in muscle correlates with amyotrophic lateral sclerosis severity. Annals of Neurology.

[CR41] Josephson A, Widenfalk J, Widmer HW, Olson L, Spenger C (2001). NOGO mRNA expression in adult and fetal human and rat nervous tissue and in weight drop injury. Experimental Neurology.

[CR42] Karnezis T, Mandemakers W, McQualter JL, Zheng B, Ho PP, Jordan KA (2004). The neurite outgrowth inhibitor Nogo A is involved in autoimmune-mediated demyelination. Nature Neuroscience.

[CR43] Kaya L, Meissner B, Riedl MC, Muik M, Schwarzer C, Ferraguti F (2013). Direct association of the reticulon protein RTN1A with the ryanodine receptor 2 in neurons. Biochimica et Biophysica Acta.

[CR44] Kim JE, Li S, GrandPré T, Qiu D, Strittmatter SM (2003). Axon regeneration in young adult mice lacking Nogo-A/B. Neuron.

[CR45] Kuang E, Wan Q, Li X, Xu H, Liu Q, Qi Y (2005). ER Ca^2+^ depletion triggers apoptotic signals for endoplasmic reticulum (ER) overload response induced by overexpressed reticulon 3 (RTN3/HAP). Journal of Cellular Physiology.

[CR46] Kuang E, Wan Q, Li X, Xu H, Zou T, Qi Y (2006). ER stress triggers apoptosis induced by Nogo-B/ASY overexpression. Experimental Cell Research.

[CR47] Kumamaru E, Kuo CH, Fujimoto T, Kohama K, Zeng LH, Taira E (2004). Reticulon3 expression in rat optic and olfactory systems. Neuroscience Letters.

[CR48] Kume H, Konishi Y, Murayama KS, Kametani F, Araki W (2009). Expression of reticulon 3 in Alzheimer’s disease brain. Neuropathology and Applied Neurobiology.

[CR49] Kume H, Murayama KS, Araki W (2009). The two-hydrophobic domain tertiary structure of reticulon proteins is critical for modulation of beta-secretase BACE1. Journal of Neuroscience Research.

[CR50] Lee JT, Lee TJ, Kim CH, Kim NS, Kwon TK (2009). Over-expression of Reticulon 3 (RTN3) enhances TRAIL-mediated apoptosis via up-regulation of death receptor 5 (DR5) and down-regulation of c-FLIP. Cancer Letters.

[CR51] Lee JT, Petratos S (2013). Multiple sclerosis: does Nogo play a role?. Neuroscientist.

[CR52] Li M, Song J (2007). The N- and C-termini of the human Nogo molecules are intrinsically unstructured: bioinformatics, CD, NMR characterization, and functional implications. Proteins.

[CR53] Liu TY, Bian X, Sun S, Hu X, Klemm RW, Prinz WA (2012). Lipid interaction of the C terminus and association of the transmembrane segments facilitate atlastin-mediated homotypic endoplasmic reticulum fusion. Proceedings of the National Academy of Sciences of the United States of America.

[CR54] Liu Y, Vidensky S, Ruggiero AM, Maier S, Sitte HH, Rothstein JD (2008). Reticulon RTN2B regulates trafficking and function of neuronal glutamate transporter EAAC1. The Journal of Biological Chemistry.

[CR55] Lumb JH, Connell JW, Allison R, Reid E (2012). The AAA ATPase spastin links microtubule severing membrane modelling. Biochimica et Biophysica Acta.

[CR56] Mannan AU, Boehm J, Sauter SM, Rauber A, Byrne PC, Neesen J (2006). Spastin, the most commonly mutated protein in hereditary spastic paraplegia interacts with Reticulon 1 an endoplasmic reticulum protein. Neurogenetics.

[CR57] Mannan AU, Krawen P, Sauter SM, Boehm J, Chronowska A, Paulus W (2006). ZFYVE27 (SPG33), a novel spastin-binding protein, is mutated in hereditary spastic paraplegia. The American Journal of Human Genetic.

[CR58] Melino S, Nepravishta R, Bellomaria A, Di Marco S, Paci M (2009). Nucleic acid binding of the RTN1-C C-terminal region: Toward the functional role of a reticulon protein. Biochemistry.

[CR5900] Mi, S., Hu, B., Hahm, K., Luo, Y., Kam Hui, E. S., Yuan, Q., et al. (2007). LINGO-1 antagonist promotes spinal cord remyelination and axonal integrity in MOG-induced experimental autoimmune encephalomyelitis. *Nature Medicine, 13*(10), 1228–1233. 10.1038/nm1664 17906634

[CR590] Mi, S., Miller, R. H., Lee, X., Scott, M. L., Shulag-Morskaya, S., Shao, Z., et al. (2005). LINGO-1 negatively regulates myelination by oligodendrocytes. *Nature Neuroscience, 8*(6), 745–751. 10.1038/nn1460 15895088

[CR59] Mitchell JD, Borasio GD (2007). Amyotrophic laterl sclerosis. Lancet.

[CR60] Montenegro G, Rebelo AP, Connell J, Allison R, Babalini C, D’Aloia M (2012). Mutations in the ER-shaping protein reticulon 2 cause the axon-degenerative disorder hereditary spastic paraplegia type 12. The Journal of Clinical Investigation.

[CR61] Moreira EF, Jaworski CJ, Rodriguez IR (1999). Cloning of a novel member of the reticulon gene family (RTN3): Gene structure and chromosomal localization to 11q13. Genomics.

[CR62] Morin-Leisk J, Saini SG, Meng X, Makhov AM, Zhang P, Lee TH (2011). An intramolecular salt bridge drives the soluble domain of GTP-bound atlastin into the postfusion conformation. The Journal of Cell Biology.

[CR63] Moss TJ, Andreazza C, Verma A, Daga A, McNew JA (2011). Membrane fusion by the GTPase atlastin requires a conserved C-terminal cytoplasmic tail and dimerization through the middle domain. Proceedings of the National Academy of Sciences of the United States of America.

[CR64] Murayama KS, Kametani F, Saito S, Kume H, Akiyama H, Araki W (2006). Reticulons RTN3 and RTN4-B/C interact with BACE1 and inhibit its ability to produce amyloid beta-protein. European Journal of Neuroscience.

[CR65] Nepravishta R, Bellomaria A, Polizio F, Paci M, Melino S (2010). Reticulon RTN1-C(CT) peptide: A potential nuclease and inhibitor of histone deacetylase enzymes. Biochemistry.

[CR66] Noseworthy JH, Lucchinetti C, Rodriguez M, Weinshenker BG (2000). Multiple sclerosis. The New England Journal of Medicine.

[CR67] Oertle T, Klinger M, Stuermer CA, Schwab ME (2003). A reticular rhapsody: Phylogenic evolution and nomenclature of the RTN/Nogo gene family. FASEB Journal.

[CR68] Oertle T, Merkler D, Schwab ME (2003). Do cancer cells die because of Nogo-B?. Oncogene.

[CR69] Oertle T, Schwab ME (2003). Nogo and its paRTNers. Trends in Cell Biology.

[CR70] Oertle T, van der Haar ME, Bandtlow CE, Robeva A, Burfeind P, Buss A (2003). Nogo-A inhibits neurite outgrowth and cell spreading with three discrete regions. The Journal of Neurosciences.

[CR71] O’Neill P, Whalley K, Ferretti P (2004). Nogo and Nogo-66 receptor in human and chick: Implications for development and regeneration. Developmental Dynamics.

[CR72] Orlacchio A, Kawarai T, Totaro A, Errico A, St George-Hyslop PH, Rugarli EI (2004). Hereditary spastic paraplegia: Clinical genetic study of 15 families. Archives of Neurology.

[CR73] Orso G, Pendin D, Liu S, Tosetto J, Moss TJ, Faust JE (2009). Homotypic fusion of ER membranes requires the dynamin-like GTPase atlastin. Nature.

[CR74] O’Sullivan NC, Jahn TR, Reid E, O’Kane CJ (2012). Reticulon-like-1, the Drosophila orthologue of the hereditary spastic paraplegia gene reticulon 2, is required for organization of endoplasmic reticulum and of distal motor axons. Human Molecular Genetics.

[CR75] Park SH, Blackstone C (2010). Further assembly required: Construction and dynamics of the endoplasmic reticulum network. EMBO Reports.

[CR76] Park SH, Zhu PP, Parker RL, Blackstone C (2010). Hereditary spastic paraplegia proteins REEP1, spastin, and atlastin-1 coordinate microtubule interactions with the tubular ER network. The Journal of Clinical Investigation.

[CR77] Petratos S, Ozturk E, Azari MF, Kenny R, Lee JY, Magee KA (2012). Limiting multiple sclerosis related axonopathy by blocking Nogo receptor and CRMP-2 phosphorylation. Brain.

[CR78] Pradat PF, Bruneteau G, Gonzalez de Aguilar JL, Dupuis L, Jokic N, Salachas F (2007). Muscle Nogo-A expression is a prognostic marker in lower motor neuron syndromes. Annals of Neurology.

[CR79] Prinjha R, Moore SE, Vinson M, Blake S, Morrow R, Christie G (2000). Inhibitor of neurite outgrowth in humans. Nature.

[CR80] Qi B, Qi Y, Watari A, Yoshioka N, Inoue H, Minemoto Y (2003). Pro-apoptotic ASY/Nogo-B protein associates with ASYIP. Journal of Cellular Physiology.

[CR81] Reindl M, Khantane S, Ehling R, Schanda K, Lutterotti A, Brinkhoff C (2003). Serum and cerebrospinal fluid antibodies to Nogo-A in patients with multiple sclerosis and acute neurological disorders. Journal of Neuroimmunology.

[CR82] Rezai-Zadeh K, Gate D, Town T (2009). CNS infiltration of peripheral immune cells: D-Day for neurodegenerative disease?. Journal of NeuroImmune Pharmacology.

[CR83] Roebroek AJ, Contreras B, Pauli IG, Van de Ven WJ (1998). cDNA cloning, genomic organization, and expression of the human RTN2 gene, a member of a gene family encoding reticulons. Genomics.

[CR84] Roebroek AJ, van de Velde HJ, van Bokhoven A, Broers JL, Ramaekers FC, Van de Ven WJ (1993). Cloning and expression of alternative transcripts of a novel neuroendocrine-specific gene and identification of its 135-kDa translational product. Journal of Biological Chemistry.

[CR85] Sanderson CM, Connell JW, Edwards TL, Bright NA, Duley S, Thompson A (2006). Spastin and atlastin, two proteins mutated in autosomal-dominant hereditary spastic paraplegia, are binding partners. Human Molecular Genetics.

[CR86] Satoh J, Onoue H, Arima K, Yamamura T (2005). Nogo-A and nogo receptor expression in demyelinating lesions of multiple sclerosis. Journal of Neuropathology and Experimental Neurology.

[CR87] Schanda K, Hermann M, Stefanova N, Gredler V, Bandtlow C, Reindl M (2011). Nogo-B is associated with cytoskeletal structures in human monocyte-derived macrophages. BMC Research Notes.

[CR88] Schwab ME (2010). Functions of Nogo proteins and their receptors in the nervous system. Nature Reviews Neuroscience.

[CR89] Schwartz M, London A, Shechter R (2009). Boosting T-cell immunity as a therapeutic approach for neurodegenerative conditions: The role of innate immunity. Neuroscience.

[CR90] Schweigreiter R, Stasyk T, Contarini I, Frauscher S, Oertle T, Klimaschewski L (2007). Phosphorylation-regulated cleavage of the reticulon protein Nogo-B by caspase-7 at a noncanonical recognition site. Proteomics.

[CR91] Shao Z, Browning JL, Lee X, Scott ML, Shulga-Morskaya S, Allaire N (2005). TAJ/TROY, an orphan TNF receptor family member, binds Nogo-66 receptor 1 and regulates axonal regeneration. Neuron.

[CR92] Shi Q, Prior M, He W, Tang X, Hu X, Yan R (2009). Reduced amyloid deposition in mice overexpressing RTN3 is adversely affected by preformed dystrophic neurites. The Journal of Neuroscience.

[CR93] Shi Q, Prior M, Zhou X, Tang X, He W, Hu X (2013). Preventing formation of reticulon 3 immunoreactive dystrophic neurites improves cognitive function in mice. The Journal of Neuroscience.

[CR94] Shibata Y, Hu J, Kozlov MM, Rapoport TA (2009). Mechanisms shaping the membranes of cellular organelles. Annual Review of Cell and Developmental Biology.

[CR95] Shibata Y, Shemesh T, Prinz WA, Palazzo AF, Kozlov MM, Rapoport TA (2010). Mechanisms determining the morphology of the peripheral ER. Cell.

[CR96] Simonen M, Pedersen V, Weinmann O, Schnell L, Buss A, Ledermann B (2003). Systemic deletion of the myelin-associated outgrowth inhibitor Nogo-A improves regenerative and plastic responses after spinal cord injury. Neuron.

[CR970] Small, S. A., & Gandy, S. (2006). Sorting through the cell biology of Alzheimer’s disease: Intracellular pathways to pathogenesis. *Neuron, 52*(1), 15–31. 10.1016/j.neuron.2006.09.001 PMC4820242 17015224

[CR97] Sparkes I, Tolley N, Aller I, Osterrieder A, Botchway S, Mueller C (2010). Five Arabidopsis reticulon isoforms share endoplasmic reticulum location, topology, and membrane-shaping properties. The Plant Cell.

[CR98] Steiner P, Kulangara K, Sarria JC, Glauser L, Regazzi R, Hirling H (2004). Reticulon 1-C/neuroendocrine-specific protein-C interacts with SNARE proteins. Journal of Neurochemistry.

[CR99] Tagami S, Eguchi Y, Kinoshita M, Takeda M, Tsujimoto Y (2000). A novel protein, RTN-XS, interacts with both Bcl-XL and Bcl-2 on endoplasmic reticulum and reduces their anti-apoptotic activity. Oncogene.

[CR100] Tågerud S, Libelius R, Magnusson C (2007). Muscle Nogo-A: A marker for amyotrophic lateral sclerosis or for denervation?. Annals of Neurology.

[CR101] Tang WF, Yang SY, Wu BW, Jheng JR, Chen YL, Shih CH (2007). Reticulon 3 binds the 2C protein of enterovirus 71 and is required for viral replication. The Journal of Biological Chemistry.

[CR102] Tews B, Schönig K, Arzt ME, Clementi S, Rioult-Pedotti MS, Zemmar A (2013). Synthetic microRNA-mediated downregulation of Nogo-A in transgenic rats reveals its role as regulator of synaptic plasticity and cognitive function. Proceedings of the National Academy of Science of the United States of America.

[CR103] Tufi R, Panaretakis T, Bianchi K, Criollo A, Fazi B, Di Sano F (2008). Reduction of endoplasmic reticulum Ca^2+^ levels favors plasma membrane surface exposure of calreticulin. Cell Death and Differentiation.

[CR104] van de Velde HJ, Roebroek AJ, van Leeuwen FW, Van de Ven WJ (1994). Molecular analysis of expression in rat brain of NSP-A, a novel neuroendocrine-specific protein of the endoplasmic reticulum. Brain Research. Molecular Brain Research.

[CR105] VanGuilder Starkey HD, Bixler GV, Sonntag WE, Freeman WM (2013). Expression of NgR1-antagonizing proteins decreases with aging and cognitive decline in rat hippocampus. Cellular and Molecular Neurobiology.

[CR106] Voeltz GK, Prinz WA, Shibata Y, Rist JM, Rapoport TA (2006). A class of membrane proteins shaping the tubular endoplasmic reticulum. Cell.

[CR107] Wakana Y, Koyama S, Nakajima K, Hatsuzawa K, Nagahama M, Tani K (2005). Reticulon 3 is involved in membrane trafficking between the endoplasmic reticulum and Golgi. Biochemical and Biophysical Research Communications.

[CR108] Wälchli T, Pernet V, Weinmann O, Shiu JY, Guzik-Kornacka A, Decrey G (2013). Nogo-A is a negative regulator of CNS angiogenesis. Proceedings of the National Academy of Science of the United States of America.

[CR109] Wan Q, Kuang E, Dong W, Zhou S, Xu H, Qi Y (2007). Reticulon 3 mediates Bcl-2 accumulation in mitochondria in response to endoplasmic reticulum stress. Apoptosis.

[CR1090] Wang, K. C., Kim, J. A., Sivasankaran, R., Segal, R., & He, Z. (2002). P75 interacts with the Nogo receptor as a co-receptor for Nogo, MAG and OMgp. *Nature, 420*(6911), 74–78. 10.1038/nature01176 12422217

[CR110] Wieczoerk DF, Hughes SR (1991). Developmentally regulated cDNA expressed exclusively in neuronal tissue. Brain Research. Molecular Brain Research.

[CR111] Wojcik S, Engel WK, Askanas V (2006). Increased expression of Noga-A in ALS muscle biopsies is not unique for this disease. Archive of “Acta Myologica”.

[CR112] Wright PL, Yu J, Di YP, Homer RJ, Chupp G, Elias JA (2010). Epithelial reticulon 4B (Nogo-B) is an endogenous regulator of Th2-driven lung inflammation. The Journal of Experimental Medicine.

[CR113] Xu H, Zhou Q, Liu X, Qi YP (2006). Co-involvement of the mitochondria and endoplasmic reticulum in cell death induced by the novel ER-targeted protein HAP. Cellular & Molecular Biology Letters.

[CR114] Yang YS, Harel NY, Strittmatter SM (2009). Reticulon-4A (Nogo-A) redistributes protein disulfide isomerase to protect mice from SOD1-dependent amyotrophic lateral sclerosis. The Journal of Neuroscience.

[CR115] Yang Y, Liu Y, Wei P, Peng H, Winger R, Hussain RZ (2010). Silencing Nogo-A promotes functional recovery in demyelinating disease. Annals of Neurology.

[CR116] Yang YS, Strittmatter SM (2007). The reticulons: A family of proteins with diverse functions. Genome Biology.

[CR117] Yang J, Yu L, Bi AD, Zhao SY (2000). Assignment of the human reticulon 4 gene (RTN4) to chromosome 2p14 → 2p13 by radiation hybrid mapping. Cytogenetics and Cell Genetics.

[CR118] Yokota T, Mishra M, Akatsu H, Tani Y, Miyauchi T, Yamamoto T (2006). Brain site-specific gene expression analysis in Alzheimer’s disease patients. European Journal of Clinical Investigation.

[CR119] Yu J, Fernández-Hernando C, Suarez Y, Schleicher M, Hao Z, Wright PL (2009). Reticulon 4B (Nogo-B) is necessary for macrophage infiltration and tissue repair. Proceedings of the National Academy of Science of the United States of America.

[CR120] Zhang D, Utsumi T, Huang HC, Gao L, Sangwung P, Chung C (2011). Reticulon 4B (Nogo-B) is a novel regulator of hepatic fibrosis. Hepatology.

[CR121] Zhao B, Pan BS, Shen SW, Sun X, Hou ZZ, Yan R (2013). Diabetes-induced central neuritic dystrophy and cognitive deficits are associated with the formation of oligomeric reticulon-3 via oxidative stress. The Journal of Biological Chemistry.

[CR122] Zhu L, Xiang R, Dong W, Liu Y, Qi Y (2007). Anti-apoptotic activity of Bcl-2 is enhanced by its interaction with RTN3. Cell Biology International.

[CR123] Zurek N, Sparks L, Voeltz G (2011). Reticulon short hairpin transmembrane domains are used to shape ER tubules. Traffic.

